# Biphenyl-3,3′-dicarb­oxy­lic acid

**DOI:** 10.1107/S1600536811014334

**Published:** 2011-04-29

**Authors:** Shao-Hua Deng, Jun Zhao, Yi-Qiang Mu, Cai Li, Hui-Min Liu

**Affiliations:** a College of Mechanical and Materials Engineering, China Three Gorges University, Yichang, Hubei 443002, People’s Republic of China

## Abstract

The asymmetric unit of the title compound, C_14_H_10_O_4_, contains one half mol­ecule, the complete mol­ecule being generated by a twofold axis. The two benzene rings form a dihedral angle of 43.11 (5)°. Inter­molecular O—H⋯O hydrogen bonds link the mol­ecules into one-dimensional zigzag chains. These chains are further connected into two-dimensional supra­molecular layers by weak π–π stacking inter­actions between neighbouring benzene rings, with centroid–centroid distances of 3.7648 (8) Å.

## Related literature

For general background non-covalent intermolecular interactions, see: Etter *et al.* (1990 ▶); Desiraju (2003 ▶); Yaghi *et al.* (2003 ▶); Li *et al.* (2010 ▶). For the structures of related complexes, see: Wang *et al.* (2005 ▶); Zhu (2010 ▶).
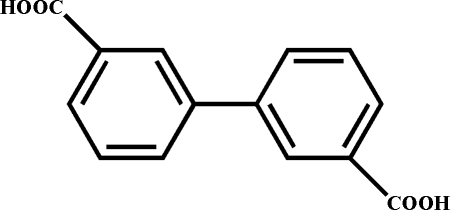


## Experimental

### 

#### Crystal data

C_14_H_10_O_4_
*M* *_r_* = 242.22
Monoclinic, 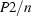

*a* = 6.6123 (9) Å
*b* = 3.7648 (8) Å
*c* = 22.554 (3) Å
β = 93.14 (2)°
*V* = 560.61 (15) Å^3^
*Z* = 2
Mo *K*α radiation
μ = 0.11 mm^−1^
*T* = 296 K
0.21 × 0.18 × 0.13 mm

#### Data collection

Bruker SMART CCD diffractometer
Absorption correction: multi-scan (*SADABS*; Sheldrick, 1996 ▶) *T*_min_ = 0.978, *T*_max_ = 0.986
5212 measured reflections
1286 independent reflections
1006 reflections with *I* > 2σ(*I*)
*R*_int_ = 0.107

#### Refinement

*R*[*F* ^2^ > 2σ(*F* ^2^)] = 0.052
*wR*(*F* ^2^) = 0.155
*S* = 1.04
1286 reflections
83 parameters
H-atom parameters constrained
Δρ_max_ = 0.24 e Å^−3^
Δρ_min_ = −0.25 e Å^−3^

### 

Data collection: *SMART* (Bruker, 1997 ▶); cell refinement: *SAINT* (Bruker, 1997 ▶); data reduction: *SAINT*; program(s) used to solve structure: *SHELXS97* (Sheldrick, 2008 ▶); program(s) used to refine structure: *SHELXL97* (Sheldrick, 2008 ▶); molecular graphics: *SHELXTL* (Sheldrick, 2008 ▶); software used to prepare material for publication: *SHELXTL*.

## Supplementary Material

Crystal structure: contains datablocks I, global. DOI: 10.1107/S1600536811014334/zq2097sup1.cif

Structure factors: contains datablocks I. DOI: 10.1107/S1600536811014334/zq2097Isup2.hkl

Additional supplementary materials:  crystallographic information; 3D view; checkCIF report

## Figures and Tables

**Table 1:** Hydrogen-bond geometry (Å, °)

*D*—H⋯*A*	*D*—H	H⋯*A*	*D*⋯*A*	*D*—H⋯*A*
O1—H1⋯O2^i^	0.82	1.82	2.6268 (17)	169

Symmetry code: (i) 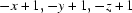.

**Fig. 1.**
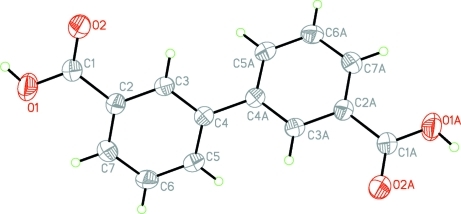
The molecular structure of the title compound, with atom labels and 50% probability displacement ellipsoids for non-H atoms [symmetry code (A): -x + 3/2, y, -z + 1/2].

**Fig. 2.**
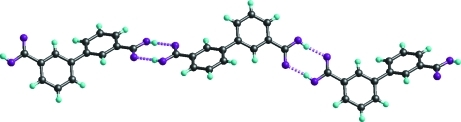
View of the one-dimensional chains connected by cyclic R22(8) hydrogen-bonds.

**Fig. 3.**
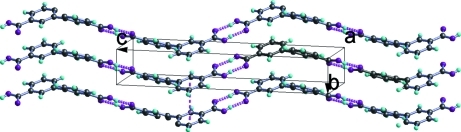
View of the two-dimensional layers formed by weak π-π stacking interactions between neighbouring benzene rings.

## supplementary crystallographic information

### Comment

Non-covalent intermolecular interactions, mainly hydrogen bonding and aromatic stacking, play the key role to perfectly project and regulate the detailed crystal packing of supramolecular materials (Desiraju, 2003). Aromatic carboxylates have also been proved to be effective building blocks in constructing various architectures (Yaghi *et al.*, 2003; Li *et al*., 2010; Wang *et al.*, 2005; Zhu, 2010). Recently, we obtained the title compound under hydrothermal conditions and we report its crystal structure here.

The asymmetric unit of the title compound, C_14_H_10_O_4_, contains one-half molecule, the complete molecule being generated by a two-fold axis (Fig. 1). The two benzene rings form a dihedral angle of 43.11 (5)°. The carboxylic acid groups form the classic cyclic *R*_2_^2^(8) hydrogen-bond motif (Etter *et al.*, 1990) with other acid groups of neighbouring molecules (Table 1). These interactions result into one-dimensional zigzag chains (Fig. 2). The chains are further connected into two-dimensional supramolecular layers by weak π-π stacking interactions between neighbouring benzene rings, with centroid-centroid distances of 3.7648 (8) Å.

### Experimental

A mixture of 3,3'-biphenyldicarboxylic acid (0.0242 g, 0.1 mmol), Pb(CH_3_COO)_2_ (0.0379 g, 0.1 mmol), water (8 ml) was stired vigorously for 30 min and then sealed in a Teflon-lined stainless-steel autoclave. The autoclave was heated and maintained at 413 K for 3 days, and then cooled to room temperature at 5 K h^-1^ to obtain colorless prism crystals suitable for X-ray analysis.

### Refinement

All H atoms were positioned geometrically (C—H = 0.93 Å and O—H = 0.82 Å) and allowed to ride on their parent atoms, with *U*_iso_(H) = 1.2 *U*_eq_(C) or *U*_iso_(H) = 1.5 *U*_eq_(O).

### Crystal data

**Table:**

C_14_H_10_O_4_	*F*(000) = 252
*M**_r_* = 242.22	*D*_x_ = 1.435 Mg m^−^^3^
Monoclinic, *P*2/*n*	Mo *K*α radiation, λ = 0.71073 Å
Hall symbol: -P 2yac	Cell parameters from 1286 reflections
*a* = 6.6123 (9) Å	θ = 3.2–27.6°
*b* = 3.7648 (8) Å	µ = 0.11 mm^−^^1^
*c* = 22.554 (3) Å	*T* = 296 K
β = 93.14 (2)°	Prism, colourless
*V* = 560.61 (15) Å^3^	0.21 × 0.18 × 0.13 mm
*Z* = 2	

### Data collection

**Table:**

Bruker SMART CCD diffractometer	1286 independent reflections
Radiation source: fine-focus sealed tube	1006 reflections with *I* > 2σ(*I*)
graphite	*R*_int_ = 0.107
φ and ω scans	θ_max_ = 27.6°, θ_min_ = 3.2°
Absorption correction: multi-scan (*SADABS*; Sheldrick, 1996)	*h* = −8→8
*T*_min_ = 0.978, *T*_max_ = 0.986	*k* = −4→4
5212 measured reflections	*l* = −29→28

### Refinement

**Table:**

Refinement on *F*^2^	Primary atom site location: structure-invariant direct methods
Least-squares matrix: full	Secondary atom site location: difference Fourier map
*R*[*F*^2^ > 2σ(*F*^2^)] = 0.052	Hydrogen site location: inferred from neighbouring sites
*wR*(*F*^2^) = 0.155	H-atom parameters constrained
*S* = 1.04	*w* = 1/[σ^2^(*F*_o_^2^) + (0.0843*P*)^2^] where *P* = (*F*_o_^2^ + 2*F*_c_^2^)/3
1286 reflections	(Δ/σ)_max_ < 0.001
83 parameters	Δρ_max_ = 0.24 e Å^−^^3^
0 restraints	Δρ_min_ = −0.25 e Å^−^^3^

### Special details

**Table:**

Geometry. All e.s.d.'s (except the e.s.d. in the dihedral angle between two l.s. planes) are estimated using the full covariance matrix. The cell e.s.d.'s are taken into account individually in the estimation of e.s.d.'s in distances, angles and torsion angles; correlations between e.s.d.'s in cell parameters are only used when they are defined by crystal symmetry. An approximate (isotropic) treatment of cell e.s.d.'s is used for estimating e.s.d.'s involving l.s. planes.
Refinement. Refinement of *F*^2^ against ALL reflections. The weighted *R*-factor *wR* and goodness of fit *S* are based on *F*^2^, conventional *R*-factors *R* are based on *F*, with *F* set to zero for negative *F*^2^. The threshold expression of *F*^2^ > σ(*F*^2^) is used only for calculating *R*-factors(gt) *etc*. and is not relevant to the choice of reflections for refinement. *R*-factors based on *F*^2^ are statistically about twice as large as those based on *F*, and *R*- factors based on ALL data will be even larger.

### Fractional atomic coordinates and isotropic or equivalent isotropic displacement parameters (Å^2^)

**Table:**

	*x*	*y*	*z*	*U*_iso_*/*U*_eq_	
O1	0.73331 (19)	0.2984 (4)	0.48869 (5)	0.0617 (5)	
H1	0.6601	0.3923	0.5124	0.093*	
C2	0.7741 (2)	0.2032 (4)	0.38631 (6)	0.0340 (4)	
O2	0.48155 (17)	0.4652 (4)	0.42503 (5)	0.0527 (4)	
C3	0.6866 (2)	0.2063 (4)	0.32849 (6)	0.0323 (4)	
H3	0.5542	0.2860	0.3216	0.039*	
C4	0.79643 (19)	0.0906 (4)	0.28086 (6)	0.0321 (4)	
C1	0.6531 (2)	0.3318 (4)	0.43585 (6)	0.0373 (4)	
C5	0.9952 (2)	−0.0282 (4)	0.29269 (7)	0.0384 (4)	
H5	1.0701	−0.1063	0.2615	0.046*	
C7	0.9722 (2)	0.0838 (4)	0.39736 (7)	0.0402 (4)	
H7	1.0301	0.0811	0.4359	0.048*	
C6	1.0825 (2)	−0.0316 (4)	0.35005 (8)	0.0421 (4)	
H6	1.2149	−0.1110	0.3570	0.050*	

### Atomic displacement parameters (Å^2^)

**Table:**

	*U*^11^	*U*^22^	*U*^33^	*U*^12^	*U*^13^	*U*^23^
O1	0.0573 (8)	0.1005 (11)	0.0274 (6)	0.0284 (7)	0.0010 (5)	−0.0027 (6)
C2	0.0336 (7)	0.0401 (7)	0.0285 (7)	0.0010 (6)	0.0038 (5)	0.0021 (6)
O2	0.0415 (7)	0.0832 (9)	0.0336 (6)	0.0178 (6)	0.0024 (5)	−0.0019 (5)
C3	0.0270 (7)	0.0397 (7)	0.0305 (7)	0.0014 (5)	0.0030 (5)	0.0024 (5)
C4	0.0297 (7)	0.0374 (7)	0.0295 (8)	−0.0018 (5)	0.0033 (5)	0.0013 (5)
C1	0.0352 (8)	0.0475 (8)	0.0291 (7)	0.0020 (6)	0.0012 (5)	0.0009 (6)
C5	0.0294 (7)	0.0501 (9)	0.0363 (8)	0.0027 (6)	0.0071 (6)	−0.0010 (6)
C7	0.0350 (8)	0.0520 (9)	0.0332 (8)	0.0019 (6)	−0.0028 (5)	0.0037 (6)
C6	0.0272 (7)	0.0571 (9)	0.0418 (9)	0.0075 (6)	0.0014 (6)	0.0046 (7)

### Geometric parameters (Å, °)

**Table:**

O1—C1	1.2837 (17)	C4—C5	1.400 (2)
O1—H1	0.8200	C4—C4^i^	1.490 (3)
C2—C7	1.394 (2)	C5—C6	1.388 (2)
C2—C3	1.3975 (19)	C5—H5	0.9300
C2—C1	1.490 (2)	C7—C6	1.394 (2)
O2—C1	1.2520 (18)	C7—H7	0.9300
C3—C4	1.399 (2)	C6—H6	0.9300
C3—H3	0.9300		
			
C1—O1—H1	109.5	O2—C1—C2	120.13 (13)
C7—C2—C3	120.43 (13)	O1—C1—C2	116.90 (12)
C7—C2—C1	120.56 (13)	C6—C5—C4	121.26 (13)
C3—C2—C1	119.01 (12)	C6—C5—H5	119.4
C2—C3—C4	120.53 (12)	C4—C5—H5	119.4
C2—C3—H3	119.7	C2—C7—C6	119.28 (14)
C4—C3—H3	119.7	C2—C7—H7	120.4
C3—C4—C5	118.33 (13)	C6—C7—H7	120.4
C3—C4—C4^i^	120.83 (14)	C5—C6—C7	120.16 (13)
C5—C4—C4^i^	120.83 (14)	C5—C6—H6	119.9
O2—C1—O1	122.97 (13)	C7—C6—H6	119.9
			
C7—C2—C3—C4	−0.1 (2)	C3—C2—C1—O1	174.69 (14)
C1—C2—C3—C4	179.39 (13)	C3—C4—C5—C6	−0.1 (2)
C2—C3—C4—C5	0.1 (2)	C4^i^—C4—C5—C6	−179.54 (11)
C2—C3—C4—C4^i^	179.53 (10)	C3—C2—C7—C6	0.2 (2)
C7—C2—C1—O2	174.22 (14)	C1—C2—C7—C6	−179.33 (14)
C3—C2—C1—O2	−5.3 (2)	C4—C5—C6—C7	0.2 (2)
C7—C2—C1—O1	−5.8 (2)	C2—C7—C6—C5	−0.2 (2)

Symmetry codes: (i) −*x*+3/2, *y*, −*z*+1/2.

### Hydrogen-bond geometry (Å, °)

**Table:**

*D*—H···*A*	*D*—H	H···*A*	*D*···*A*	*D*—H···*A*
O1—H1···O2^ii^	0.82	1.82	2.6268 (17)	169.

Symmetry codes: (ii) −*x*+1, −*y*+1, −*z*+1.

## References

[bb1] Bruker (1997). *SMART* and *SAINT.* Bruker AXS Inc., Madison, Wisconsin, USA.

[bb2] Desiraju, G. R. (2003). *J. Mol. Struct.* **656**, 5–15.

[bb3] Etter, M. C., MacDonald, J. C. & Bernstein, J. (1990). *Acta Cryst.* B**46**, 256–262. 10.1107/s0108768189012929 2344397

[bb4] Li, D. S., Wu, Y. P., Zhang, P., Du, M., Zhao, J., Li, C. P. & Wang, Y. Y. (2010). *Cryst. Growth Des.* **10**, 2037–2040.

[bb5] Sheldrick, G. M. (1996). *SADABS* University of Göttingen, Germany.

[bb6] Sheldrick, G. M. (2008). *Acta Cryst.* A**64**, 112–122. 10.1107/S0108767307043930 18156677

[bb7] Wang, R. H., Han, L., Jiang, F. L., Zhou, Y. F., Yuan, D. Q. & Hong, M. C. (2005). *Cryst. Growth Des.* **5**, 129–135.

[bb8] Yaghi, O. M., O’Keeffe, M., Ockwing, N. W., Chae, H. K., Eddaoudi, M. & Kim, J. (2003). *Nature* *(London)*, **423**, 705–714. 10.1038/nature01650 12802325

[bb9] Zhu, B.-Y. (2010). *Acta Cryst.* E**66**, m1214. 10.1107/S1600536810035269 PMC2983367 21587372

